# Embryonic Programs in Cancer and Metastasis—Insights From the Mammary Gland

**DOI:** 10.3389/fcell.2022.938625

**Published:** 2022-06-29

**Authors:** May Yin Lee

**Affiliations:** Genome Institute of Singapore, Agency for Science, Technology and Research, Singapore, Singapore

**Keywords:** mammary gland, embryonic development, breast cancer, metastasis, molecular signatures

## Abstract

Cancer is characterized as a reversion of a differentiated cell to a primitive cell state that recapitulates, in many aspects, features of embryonic cells. This review explores the current knowledge of developmental mechanisms that are essential for embryonic mouse mammary gland development, with a particular focus on genes and signaling pathway components that are essential for the induction, morphogenesis, and lineage specification of the mammary gland. The roles of these same genes and signaling pathways in mammary gland or breast tumorigenesis and metastasis are then summarized. Strikingly, key embryonic developmental pathways are often reactivated or dysregulated during tumorigenesis and metastasis in processes such as aberrant proliferation, epithelial-to-mesenchymal transition (EMT), and stem cell potency which affects cellular lineage hierarchy. These observations are in line with findings from recent studies using lineage tracing as well as bulk- and single-cell transcriptomics that have uncovered features of embryonic cells in cancer and metastasis through the identification of cell types, cell states and characterisation of their dynamic changes. Given the many overlapping features and similarities of the molecular signatures of normal development and cancer, embryonic molecular signatures could be useful prognostic markers for cancer. In this way, the study of embryonic development will continue to complement the understanding of the mechanisms of cancer and aid in the discovery of novel therapeutic targets and strategies.

## Graphical Abstract

Shared molecular programs in embryonic mammary gland cells and breast cancers. Key developmental pathways are often reactivated or dysregulated during tumorigenesis and metastagenesis. The identification of embryonic signatures in tumors and metastasis could pinpoint novel prognostic markers and targets for therapeutic intervention. Figure created with BioRender.
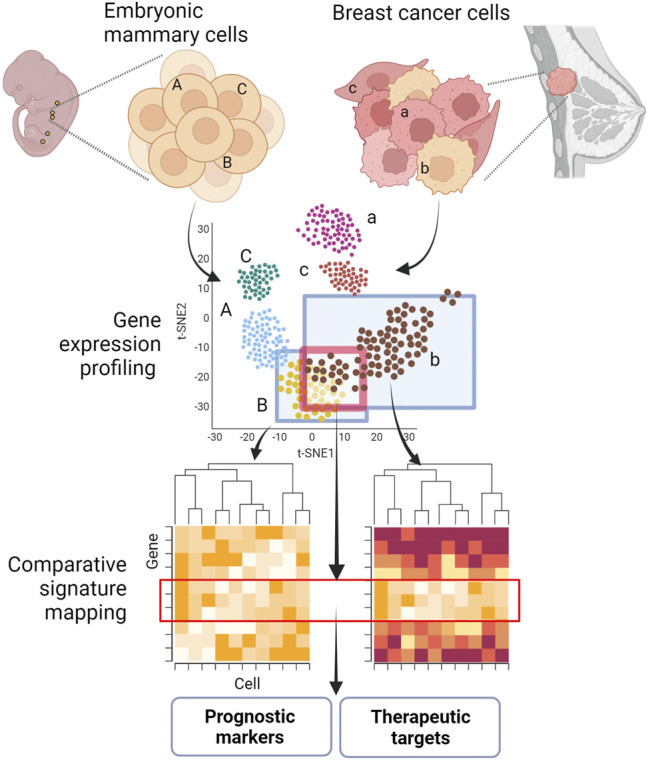

## Introduction

The mammary gland is the definitive feature of species in the class of Mammalia. Development of the mammary glands starts in the mouse embryo at embryonic (E) day 10.5 with the specification of the mammary line. By E11.75, all five mammary rudiment (MR) pairs in the mouse are present as disk-shaped, multi-layered placodal structures that will grow and acquire, sequentially, a morphology characterized by hillock, bud and bulb. Conventionally, the MR pairs are numbered 1 to 5 by their position along the antero-posterior axis. At E14.5, MR development diverges between the two sexes in a process called sexual dimorphism. MR development halts in males but proceeds in females with bulb enlargement and its recession into the mesenchyme. At E18.5, just before birth, the MR consists of a rudimentary ductal tree structure of 10–15 branches embedded within the mammary fat pad (Veltmaat et al., 2003; Spina and Cowin, 2021). The MRs develop asynchronously in the order of MR3, MR4, MR1 and MR5 and finally MR2, as determined by histological examination (Lee et al., 2011).

**FIGURE 1:**
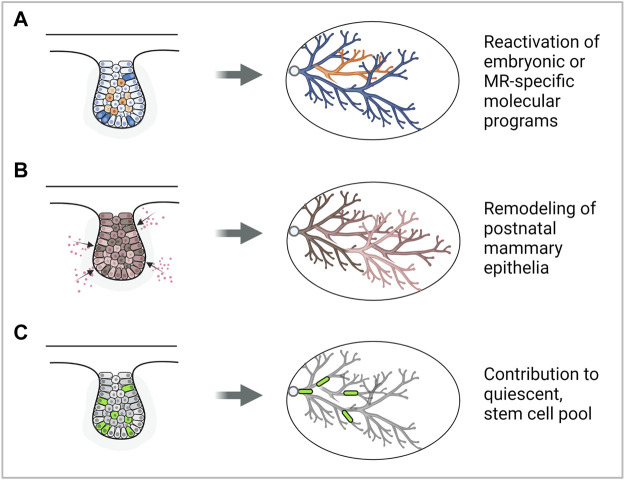
Modes by which the embryonic mammary gland may contribute to breast cancer development. **(A)** Reactivation of embryonic mammary gland genes or signaling pathways may promote cancer development. **(B)** Exposure to carcinogens may remodel the postnatal mammary gland and increase breast cancer risk. **(C)** Embryonic mammary gland cells may contribute to the stem cell pool in the postnatal mammary gland which may be cells of origin of cancer. Note: a representative MR is depicted, however, each mode could be plausibly applied to MRs in other stages of development. Figure created with BioRender.

As a skin appendage, the murine embryonic mammary glands are excellent models for understanding developmental processes such as ectodermal specification, epithelial-mesenchymal cross-talks, morphogenesis, and their underlying cellular and molecular mechanisms. Various spontaneous mouse mutants and genetically engineered mouse models (GEMMs) have greatly facilitated the discovery of genes and signaling pathways that regulate mammary gland development (Veltmaat, 2017). This deeper understanding of developmental mechanisms has also proffered new perspectives regarding pathological conditions such as cancer. Indeed, the phases in embryonic mammary gland development resemble the phases of tumorigenesis and cancer progression. For example, the induction of the embryonic MR at around E11.5 and subsequent growth mediated by ectodermal cell recruitment (Lee et al., 2011) could be likened to cellular transformation and the development of carcinoma *in situ*. Further, MR sprouting at E15.5 and branching morphogenesis at E18.5 resemble the invasion of the basement membrane and tumor stroma during the metastatic cascade. Intriguingly, these embryonic morphogenetic events that mimic stages of tumorigenesis and metastasis also share similarities at the molecular level. Collectively, insight into these associations raises the possibility that cancer cells may leverage upon early developmental pathways and molecular programs to spur pathogenic development. Reversion to a more undifferentiated, embryonic-like state may promote processes that are associated with malignancy such as proliferation, epithelial-to-mesenchymal transition (EMT), cancer stem cell (CSC) formation, invasion, and metastasis (Kelleher et al., 2006; Takebe et al., 2011; Lee et al., 2019) (Figure 1A).

Does embryonic developmental history affect postnatal development and susceptibility to cancer? Development of the MRs during embryogenesis may lay the foundation for growth and morphogenesis during the postnatal phase, which is subsequently mirrored during neoplastic transformation. It has been observed that the postnatal thoracic mammary glands (MGs 1, 2, and 3) tend to form mammary tumors more frequently than inguinal glands (MGs 4 and 5) (Sheldon et al., 1982; Minasian, 1983; Bolander, 1990). This has been attributed to the presence of more epithelial tissue in the thoracic glands compared to the inguinal glands that can undergo neoplastic transformation (Vaage, 1984). Moreover, the asynchronous differentiation between the thoracic and inguinal mammary glands results in increased less-differentiated structures such as the terminal end buds in the thoracic glands. This may explain the thoracic glands’ increased susceptibility to 7,12-dimethylbenz(a)anthracene (DMBA)-induced carcinogenesis (Russo and Russo, 1988). These observations are in line with studies showing that the mouse mammary tumor virus long terminal repeat-driven Polyoma virus middle T antigen (MMTV- PyMT) and MMTV-cNeu mouse models generate more tumors in specific thoracic mammary glands than inguinal ones (Veltmaat et al., 2013) (Figure 1B). Lastly, embryonic mammary gland development is adversely affected by its exposure to endogenous and synthetic estrogens such as Bisphenol-A (BPA) which is linked to increased breast cancer risk during adulthood (Acevedo et al., 2013; Speroni et al., 2017) (Figure 1B). Taken together, physiological differences that are associated with differential tumorigenic potential could, in part, be attributed to differential molecular regulation that has taken place in embryonic development.

The approach of seeking to understand pathological conditions from the study of normal development has provided new perspectives into the origins of cancer. Some suggest that breast cancer may have a stem cell origin as the transcription factors that normally regulate gene expression in embryonic stem or progenitor cells are also misregulated in breast cancers (Briegel, 2006). Moreover, lineage tracing studies show that proliferating, long label-retaining embryonic cells (Boras-Granic et al., 2014) and a *Lgr5* ^*+*^ *Tspan8* ^*hi*^ subpopulation (Fu et al., 2017) may contribute to the population of long-lived, quiescent mammary stem cells, which may be precursor cancer cells in the adult (Figure 1C). These findings imply the potential to simplify and deconvolute the study of tumors and their significant cellular and molecular heterogeneity by considering and focusing on subpopulations of cells having embryonic origins or molecular signatures.

This review provides a comprehensive summary of all known genes and signaling pathways that lead to aberrant embryonic mammary gland phenotypes in GEMMs as well as breast cancers and metastasis when dysregulated. Following this, recent studies that compare molecular signatures of the embryonic mammary glands and breast cancers are summarized. Evidence is provided to support the proposal that studying the development of the mammary glands aligns with providing an understanding of the mechanisms of cancer, with the intention to identify novel prognostic markers and therapeutic strategies against this prominent disease.

## Signaling Pathways in Embryonic Mammary Gland Development and Cancer

### WNT Signaling

WNT signaling regulates numerous developmental processes. The name “WNT” is a combination of wingless and integration site-1 (Int1), of which the latter was identified as a genetic locus activated by the insertion of the MMTV that leads to the formation of mammary tumors (Nusse and Varmus, 1982). Presently, there are 19 known WNT ligands in mammals. Classical WNTs (WNT1, WNT3A, WNT8, and WNT8B) activate the canonical β-catenin pathway while non-classical WNTs (WNT4, WNT5A, and WNT11) activate the non-canonical WNT/calcium pathway.

**FIGURE 2:**
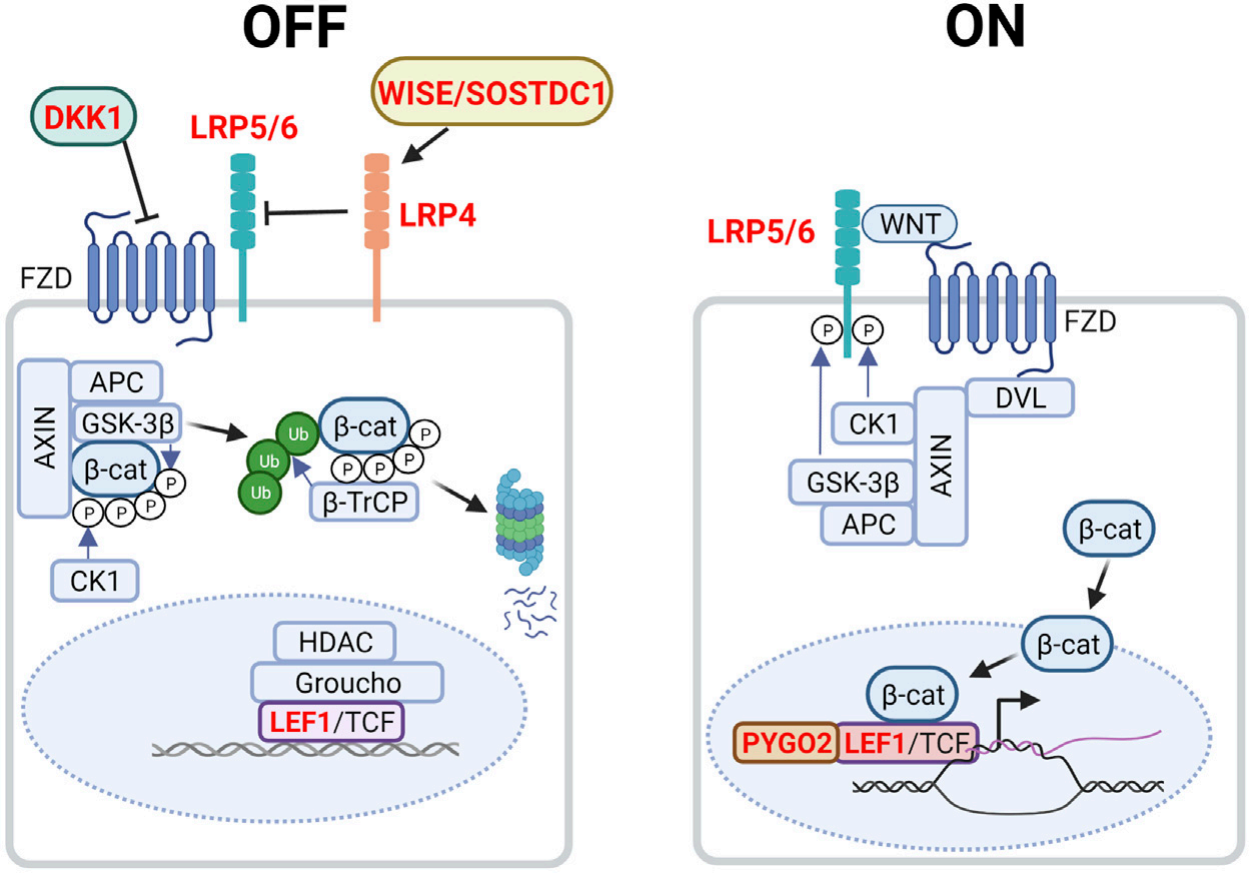
Canonical WNT/β-catenin signaling in the embryonic mammary gland. In the absence of WNT (left, OFF state), cytoplasmic β-catenin is targeted to the destruction complex comprising AXIN, APC, GSK3β and CK1 where it is phosphorylated. Phosphorylated β-catenin is ubiquitinated by the E3 ubiquitin ligase β-TrCP, which targets β-catenin for proteasomal degradation. WNT target genes are repressed by Groucho and histone deacetylases (HDACs). LRP4 and its potential ligand, WISE/SOSTDC1 inhibits WNT signaling. In the presence of WNT ligand (right, ON state), a receptor complex forms between FZD and LRP5/6. DVL recruitment by FZD leads to LRP5/6 phosphorylation and AXIN recruitment. Consequently, degradation of β-catenin is disrupted, allowing β-catenin to accumulate in the nucleus where it functions as a co-activator of LEF1/TCF to promote the transcription of target genes. Genes highlighted in red denote those that give rise to aberrant embryonic mammary gland phenotypes when deleted or overexpressed. P denotes phosphorylation events after overexpressed. Figure created with BioRender.

The canonical WNT/β-catenin pathway primarily acts to regulate cytosolic β-catenin levels (Figure 2). Without WNT, β-catenin is targeted to the APC/AXIN destruction complex where it is phosphorylated by CK1 and GSK3β (Kimelman and Xu, 2006). Consequently, phosphorylated β-catenin is ubiquitinated by the E3 ubiquitin ligase β-TrCP and targeted for proteasomal degradation. In the absence of WNT, LEF/TCF transcription factors bind to WNT response elements, enabling the recruitment of co-repressors such as Groucho and histone deacetylases (HDACs) to promote chromatin compaction and inhibit target gene transcription. In the presence of WNT however, DVL recruitment by the frizzled receptor (FZD) results in the phosphorylation of co-receptors low-density lipoprotein receptor-related proteins 5 and 6 (LRP5/6) and AXIN recruitment. This inhibits the AXIN-mediated phosphorylation and degradation of β-catenin, resulting in its accumulation and nuclear import. Nuclear β-catenin binds to LEF/TCF, replaces transcriptional repressors and recruits members of the switch/sucrose non-fermentable (SWI/SNF) family of transcriptional coactivators and other chromatin remodellers. Transcriptional co-activators BCL9/LGL and pygopus (PYGO) aid in the transport of these proteins to the TCF/β-catenin complex. As the chromatin becomes less compacted and consequently more accessible, the transcription of WNT-target genes will proceed (Nusse and Clevers, 2017) (Figure 2).

WNT signaling plays critical roles in embryonic mammary gland development as inhibition and dysregulation of multiple pathway components results in aberrant phenotypes (Table 1, Figure 2). The absolute requirement for WNT signaling is demonstrated by the failure of MR induction caused by the expression of the secreted WNT inhibitor, Dickkopf-1 (*Dkk1*) under the ectoderm- and epithelium-specific *Krt5* promoter (Chu, Hens et al., 2004). In *Lef1* ^*−/−*^ embryos all MRs regress by E15.5, with MR2 and MR3 being the first to regress shortly after induction, highlighting the requirement of WNT signaling for MR maintenance (van Genderen et al., 1994; Boras-Granic et al., 2006). *Lrp5* ^*−/−*^ and *Lrp6* ^*−/−*^ embryos form hypoplastic MRs. Additionally, *Lrp6* ^*−/−*^ embryos display defects in branching morphogenesis (Lindvall et al., 2006; Lindvall et al., 2009). *Lrp4* ^*mdig/mdig*^ (*Lrp4* hypomorph) embryos display a delay in MR initiation as well as an aberrant number and distribution of mammary precursor cells leading to abnormal morphology, number and positioning of the MRs. In contrast to the previous WNT signaling-associated mutants, the *Lrp4* ^*mdig/mdig*^ mammary defects are associated with abnormally elevated WNT/β-catenin signaling. In support of this, *Lrp4* ^*mdig/mdig*^ mammary defects are abrogated by heterozygous or homozygous-null alleles of *Lrp5* and *Lrp6*, as well as the deletion of ectoderm and MR-specific β-catenin with the *Krt14-Cre* promoter (*Krt14-Cre*;*β-catenin* ^*flox/flox*^) (Ahn et al., 2013). *Lrp4* interacts with *Wise*/*Sostdc1* which modulates WNT signaling and inhibits bone morphogenetic protein (BMP) signaling (Itasaki et al., 2003; Laurikkala et al., 2003; Lintern et al., 2009). *Wise* ^*−/−*^ mice phenocopies a subset of the *Lrp4* mammary defects, including elevated WNT/β-catenin signaling whereas *Wise* overexpression reduces the number of mammary precursor cells. Finally, embryos null for *Pygo2* or lack ectoderm- and mammary epithelium-specific *Pygo2* (*Krt14-Cre;Pygo2* ^*flox/flox*^) show aberrant induction, sprouting and branching, most often in the thoracic MRs (Gu et al., 2009). Taken together, the levels of WNT/β-catenin signaling must be precisely regulated for proper MR development.

**TABLE 1:** Genes and signaling pathways critical for embryonic mammary gland development, their corresponding role in breast cancer, involvement in other cancers, and available therapeutic targets and strategies.

Signaling pathway/genes	Roles in embryonic mammary gland development	Roles in breast cancer	Other cancer types associated with gene/pathway dysregulation	Therapeutic targets/strategies
WNT signaling	Induction Chu et al. (2004); Gu et al. (2009), morphogenesis Lindvall et al. (2006); Lindvall et al. (2009); Ahn et al. (2013), MR maintenance van Genderen et al. (1994); Boras-Granic et al. (2006), branching morphogenesis Lindvall et al. (2006); Gu et al. (2009); Lindvall et al. (2009)	Tumorigenesis Zhan et al. (2017), lineage specification, stem cell potency Centonze et al. (2020), metastasis Eyre et al. (2019)	Colorectal, gastrointestinal, leukemia, melanoma Zhan et al. (2017)	Anti-FZD antibody, small molecule inhibitors Wen et al. (2020)
*Dkk1*				
*Lef1*				
*Lrp4*				
*Lrp5/6*				
*Pygo*				
*Wise*				
HH signaling	Induction Hatsell and Cowin. (2006); Lee et al. (2011), morphogenesis Lee et al. (2011)	Tumorigenesis Fiaschi et al. (2009), EMT, development and maintenance of CSCs Tanaka et al. (2009); Zhu et al. (2019), invasiveness and metastasis O'Toole et al. (2011)	Basal cell carcinoma, medulloblastoma, pancreatic, colon, ovarian, and small-cell lung carcinomas Skoda et al. (2018)	Cyclopamine, SMO inhibitors, GLI1 antagonists (GANT58 and GANT61) Kubo et al. (2004); Bhateja et al. (2019); Riobo-Del Galdo et al. (2019)
*Gli1*				
*Gli2*				
*Gli3*				
FGF signaling	Induction Mailleux et al. (2002); Veltmaat et al. (2006), morphogenesis, MR maintenance, epithelial-mesenchymal crosstalk, branching morphogenesis Mailleux et al. (2002).	Cell proliferation McLeskey et al. (1998), metastasis McLeskey et al. (1998); Turner and Grose. (2010)	Lung, pancreatic, sarcoma Wiedlocha et al. (2021)	Tyrosine kinase inhibitors (TKIs), selective TKIs of FGFRs; monoclonal antibodies (mAbs) Santolla and Maggiolini. (2020)
*Fgf10*				
*Fgfr2b*				
P190B, IRS1, IRS2, IGF1R signaling	Induction, epithelial-mesenchymal cross talk and specification Heckman et al. (2007)	P190B—tumorigenesis, metastasis Heckman-Stoddard et al. (2009); McHenry et al. (2010), IRS1-metastasis suppressor, cancer stemness Ma et al. (2006), IRS2-metastasis promoter Nagle et al. (2004)	Esophageal, endometrial, ovarian, prostate, pancreatic Leitner et al. (2022)	IGF1R signaling inhibitors (NT compounds) IGF1R mAb, small molecule tyrosine kinase inhibitors (TKIs) of IGF1R and insulin receptor, and ligand neutralising strategies Ekyalongo and Yee. (2017)
PTHRP signaling	Mammary duct formation Wysolmerski et al. (1998), nipple sheath formation Foley et al. (2001), epithelial-mesenchymal cross-talk, sexual dimorphism Wysolmerski et al. (1998); Dunbar et al. (1999); Hiremath et al. (2012)	Cell proliferation, angiogenesis, apoptosis, bone metastasis Guise et al. (2002); Li et al. (2011)	Lung, prostate, colon, clear cell renal carcinoma, etc Edwards and Johnson. (2021)	PTHRP mAb Guise et al. (1996); Li et al. (2011)
BMP signaling	Mammary line positioning Cho et al. (2006), epithelial-mesenchymal crosstalk Hens et al. (2007), ductal outgrowth Hens et al. (2007)	Cell proliferation Alarmo and Kallioniemi. (2010); Zabkiewicz et al. (2017), EMT, cancer cells stemness, metastasis Huang et al. (2017), anoikis, negative regulator of metastasis Eckhardt et al. (2020)	Lung, adrenocortical carcinoma, medulloblastoma, colorectal, prostate, pancreatic, ovarian, bladder Bach et al. (2018)	Soluble decoy receptors, neutralising antibodies, BMPR kinase inhibitors Lowery and Rosen. (2018)
*Bmp4* *Bmpr1a*				
EDA signaling	Induction Mustonen et al. (2004), sexual dimorphism, branching morphogenesis Voutilainen et al. (2012)	Tumorigenesis and squamous metaplasia, pregnancy-dependent mammary tumors Williams et al. (2022)	Melanoma Vial et al. (2019)	N.A.
NRG3	Induction Howard et al. (2005), mammary mesenchyme specification Kogata et al. (2014)	Cell proliferation Hijazi et al. (1998)	Bladder, liver, lung, ovary, prostate, etc., Ocana et al. (2016)	N.A.
NOTCH signaling	Luminal cell specification and stem cell potency Lilja et al. (2018)	Oncogene Lamy et al. (2017); Krishna et al. (2019); Nandi and Chakrabarti. (2020), metastasis Mohammadi-Yeganeh et al. (2015), interactions with the tumor microenvironment Meurette and Mehlen. (2018)	Leukemia, adenoid cystic carcinoma, glomus tumor, lymphoma, squamous cell carcinoma, small cell lung carcinoma, urothelial carcinoma, esophageal, glioma Aster et al. (2017)	γ-secretase inhibitors, mAb, bispecific antibodies (anti-DLL4/VEGF), antibody-drug conjugates Lamy et al. (2017); Krishna et al. (2019)
HOX	Induction Veltmaat et al. (2006), morphogenesis Garcia-Gasca and Spyropoulos. (2000); Satokata et al. (2000), mammary mesenchyme formation Satokata et al. (2000)	Tumorigenesis Care et al. (1998); Briegel. (2006), tumor suppression Gilbert et al. (2010), metastasis Sun et al. (2013)	Leukemia, colorectal, liver, gastrointestinal, pancreatic, etc., Li et al. (2019)	HXR9 peptides Morgan et al. (2012)
*Hoxc6*				
*Hoxd9*				
*Msx1*				
*Msx2*				
*Pax3*				
TBX	Mammary line positioning Cho et al. (2006), induction Davenport et al. (2003), MR maintenance Jerome-Majewska et al. (2005), nipple formation, branching morphogenesis Jerome-Majewska et al. (2005)	Tumorigenesis Yarosh et al. (2008), CSC formation Fillmore et al. (2010), EMT Wang et al. (2012), metastasis Rowley et al. (2004)	Pancreatic, colorectal, melanoma, endometrial, ovarian and cervical, rhabdomyosarcomas, ovarian etc Wansleben et al. (2014)	N.A.
*Tbx2*				
*Tbx3*				
GATA3	Induction, morphogenesis Asselin-Labat et al. (2007)	Tumor suppressor Asselin-Labat et al. (2011), oncogene Shan et al. (2014), luminal lineage differentiation, negative regulator of EMT Yan et al. (2010), negative regulator of metastasis Dydensborg et al. (2009); Yan et al. (2010)	Urothelial carcinomas, basal cell carcinoma, skin squamous cell carcinoma, salivary gland ductal carcinomas, pancreatic, etc Miettinen et al. (2014)	N.A.
P63	Induction (Mills et al., 1999; Yang et al., 1999)	EMT, cell motility, invasion (Lodillinsky et al., 2016; Yoh et al., 2016), stemness (Memmi et al., 2015)	Prostate, bladder, thyroid, lung, cervix (Melino, 2011)	N.A.
Hormone signaling	Sexual dimorphism	Tumorigenesis Manavathi et al. (2013), EMT, metastasis Saha Roy and Vadlamudi. (2012); Mohammadi Ghahhari et al. (2022), tumor microenvironment remodelling Bouris et al. (2015); Vella et al. (2020)	Ovarian, prostate, leukemia, lymphoma, lung, etc., Ahmad and Kumar. (2011)	Selective ER modulators (SERMs), selective ER down-regulators (SERDs), and steroidal or non-steroidal aromatase inhibitors (AIs) Siersbaek et al. (2018)

Despite there being a lack of mutations to pathway genes, an upregulation in WNT activity has been detected in most breast cancers and is linked to reduced overall survival (Zhan et al., 2017). High levels of β-catenin and its target gene, CCND1 were detected in 60% of breast cancers and correlates with poor prognosis (Lin et al., 2000). Decreased expression of the WNT inhibitory factor (WIF1) (Ai et al., 2006) and an elevated expression of WNT ligands are commonly observed in breast cancer (Xu et al., 2020). Aberrant epigenetic changes, including methylation of the APC gene promoter which could dysregulate WNT signaling has also been detected in inflammatory breast cancer (Van der Auwera et al., 2008). Importantly, WNT hyperactivity may result due to other cancer-associated mutations and aberrant activation of cancer-associated signaling pathways. For example, stabilization of β-catenin by WNT-independent pathways, such as PIN1, P53, PTEN/AKT, and NF-κB pathways, plays a significant role in breast cancer and malignant progression (Incassati et al., 2010). Intriguingly, the embryonic transcription cofactor limb-bud and heart (LBH), a direct target of WNT signalling in epithelial development, is overexpressed in the basal subtype of breast cancer (Rieger et al., 2010); this is suggestive of a WNT-mediated reversion to an embryonic-like state during tumorigenesis.

WNT signaling activation is implicated in metastasis. Cytokine signaling from the local bone microenvironment activates NF-κB and cAMP response element-binding protein (CREB) signaling in breast cancer cells, which in turn, initiates an autocrine WNT signaling loop. This leads to CSC colony formation in the bone marrow (Eyre et al., 2019). Importantly, inhibition of WNT signaling by recombinant human DKK1 or anti-FZD is sufficient to prevent metastatic colonisation in the bone. Other therapeutic strategies to inhibit WNT signaling include the use of small molecule inhibitors against the FZD receptor, destruction complex, nuclear β-catenin, and WNT ligand modifying enzymes such as Porcupine and Tankyrase (Wen et al., 2020).

### Hedgehog Signaling

**FIGURE 3:**
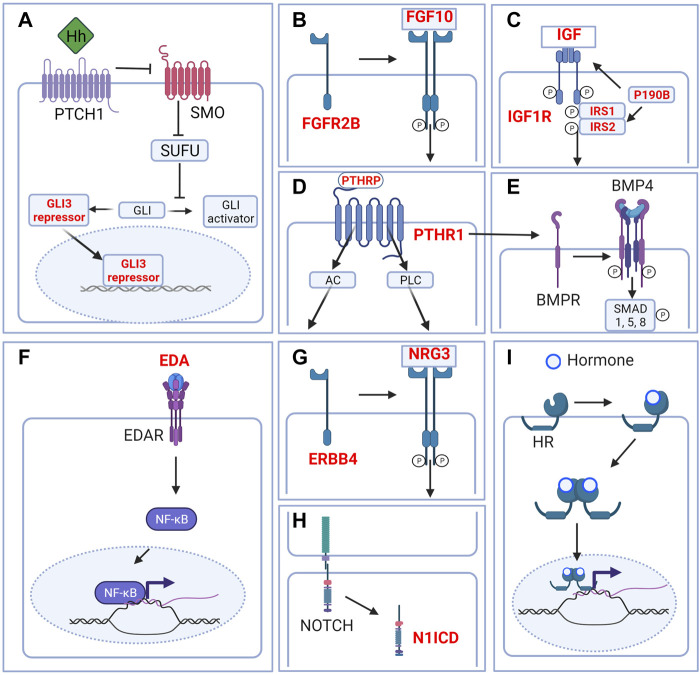
Signaling pathways in the embryonic mammary gland. Cartoon depicting major signaling pathways that are critical during embryonic mammary gland development. **(A)** Hedgehog signaling. Although Hh ligands are expressed in the MRs, Hedgehog signaling is in the inactivated state. GLI is maintained in the repressor form to repress the transcription of Hh target genes. **(B)** FGF signaling. FGF10 binding to its main receptor, FGFR2B triggers receptor dimerization, phosphorylation and the activation of diverse downstream pathways. **(C)** IGF1R signaling. Ligand binding activates the receptor kinase, leading to receptor autophosphorylation, and tyrosine phosphorylation of multiple signaling adapter proteins including, the insulin receptor substrates (IRS1/2). **(D)** PTHRP signaling. PTHRP binding to the G-coupled receptor PTHR1 activates AC and PLC downstream signaling. **(E)** BMP signaling. Binding of ligand to the receptor complex stimulates BMPR autophosphorylation and phosphorylation of downstream substrates. BMP4 may interact with PTHRP signaling to facilitate epithelial-mesenchymal cross talk. **(F)** EDA-EDAR signaling. EDA binding to EDAR triggers downstream NF-kB signaling. **(G)** NRG3-ERBB4 signaling. NRG binding triggers receptor dimerization and activation of receptor tyrosine kinase domain and downstream signaling. **(H)** NOTCH signaling. Ligand binding triggers the cleavage of N1ICD which activates downstream signaling. **(I)** Hormone signaling. Binding of hormones such as estrogen or progesterone to their cognate hormone receptor (HR) promotes internalization of the hormone-receptor complex. Homo- or heterodimer formation ensues followed by translocation into the nucleus and binding to DNA response elements and transcription or repression of target genes. Only relevant components of each signaling pathway in embryonic mammary gland development are depicted. Genes highlighted in red denote genes that give rise to aberrant embryonic mammary gland phenotypes when deleted or overexpressed. P denotes phosphorylation events. Figure created with BioRender.

The Hedgehog (HH) pathway is activated by HH ligand binding to patched (PTCH), a transmembrane transporter-like protein located at the cilium. Upon HH binding, the suppression of PTCH on the seven-span transmembrane protein smoothened (SMO) is released, leading to the release of SMO-mediated inhibition on the suppressor of fused (SUFU) (Wang et al., 2007). As a result, phosphorylation and proteolytic processing of the GLI family of zinc-finger transcription factors are inhibited and full-length proteins that function as transcriptional activators accumulate (Kise et al., 2009; Humke et al., 2010). GLI1, GLI2, and GLI3 mediate HH signaling in vertebrates; whereas GLI1 is exclusively a transcriptional activator, GLI2 and GLI3 could either activate or repress transcription (Figure 3A).

HH ligands namely Sonic hedgehog (*Shh*) and Indian hedgehog (*Ihh*) are expressed in the MRs at E12.5. Despite this, *Shh* ^*−/−*^ embryos induce all MRs and show normal branching morphogenesis at E16.5 (Michno et al., 2003). Similarly, transplantation of *Shh* ^*−/−*^ and *Ihh* ^*−/−*^ MRs into wildtype cleared fat pads results in normal branching morphogenesis. Taken together, these observations indicate that epithelial *Shh* and *Ihh* are dispensable for MR induction and branching morphogenesis (Gallego et al., 2002).

*Gli1* is a reliable marker of HH signaling activation as *Gli1* is a direct transcriptional target of HH signaling and its expression is strictly dependent on HH signaling pathway activation transduced by either *Gli2* or *Gli3* transcriptional activators (Dai et al., 1999). While *Gli1* expression is not detected in the somites underlying MR3 at E11.5, *Gli2* and more prominently, *Gli3*, are expressed. *Gli1* expression is absent in the MRs from E11.5 to E14.5. This indicates that HH signaling is inactive in the somites and MRs in these stages (Hatsell and Cowin, 2006). The upregulation of *Gli1* and, to a lesser extent, *Gli2* in *Gli3* ^*Xt-J/Xt-J*^ (*Gli3* null) MRs indicates that *Gli1* expression is suppressed by *Gli3* repressor during normal MR development. Furthermore, the loss of *Gli3* ^*Xt-J/Xt-J*^ MR3 and MR5 is phenocopied by the expression of *Gli1* under the *Gli2* promoter and *Gli3* heterozygosity (Gli2^1nki/1nki^; Gli3^Xt/+^). Altogether, this shows that HH signaling must be inactivated for MR3 and MR5 induction (Hatsell and Cowin, 2006; Lee et al., 2013). Moreover, aberrant activation of HH signaling in *Gli3* ^*Xt-J/Xt-J*^ embryos results in hypoplasia as well as defective bud and branching morphogenesis in MR2, MR4, and to a lesser extent, MR1 (Lee et al., 2011). *Gli1* ^*lzki/lzki*^ (*Gli1* null) and *Gli2* ^*lzki/lzki*^ (*Gli2* null) embryos induce all MRs normally (Hatsell and Cowin, 2006)*.*

In contrast to embryonic mammary gland development, accumulating evidence suggests that HH signaling is activated during tumorigenesis. Conditional over-expression of GLI1 with the MMTV promoter expands mammary progenitor cells, upregulates genes involved in proliferation, cell survival, EMT and metastasis and results in tumors that display the malignant basal or hybrid basal and luminal epithelial phenotypes (Fiaschi et al., 2009). Noteworthy, HH signaling components and other oncogenic pathways integrate to synergistically promote breast tumorigenesis. Specifically, GLIs may be modulated by non-HH signaling pathways through the integration of signals from TGFβ (Javelaud et al., 2011) and NF-κB (Colavito et al., 2014) signaling pathways. Similarly, although *Gli3* does not have a direct role in breast cancer, it has been found to cooperate with other genes such as androgen receptor (AR) (Lin et al., 2022), estrogen receptor (ER) (Massah et al., 2021) and *Eph10A* (Peng and Zhang, 2021) to promote tumorigenesis and invasive phenotypes. In silico analyses of gene expression profile datasets identified GLI3 as a putative interacting partner of TBX3, an important regulator in embryonic mammary gland development commonly overexpressed in breast cancer (see also section on TBX below). Further sequence-based and chromatin immunoprecipitation analyses show that *Gli3* is a direct transcriptional target of TBX3 (Mosca et al., 2009).

The HH signaling pathway is also activated in the CSC-enriched CD44^+^CD24^−/low^ population and side population of the MCF7 breast cancer cell line (Tanaka et al., 2009). The interaction of PTCH1 with the membrane glycoprotein, TSPAN8, leads to enhanced SHH signaling, increased tumor formation in mice and resistance to chemotherapeutic agents (Zhu et al., 2019). These observations suggest a link between the deregulation of HH signaling and the acquisition of cancer stemness and therapeutic resistance. Finally, in metastasis, paracrine signaling and activation of the HH pathway in stromal cells *via* tumor cell HH ligand overexpression increases invasiveness and metastasis in breast cancer (O'Toole et al., 2011).

The HH pathway has been extensively explored as therapeutic targets for breast cancer (Bhateja et al., 2019). Treatment of cyclopamine, a naturally-occurring steroidal alkaloid that inhibits the HH pathway by binding to SMO, suppresses *Gli1* expression and the growth of HH pathway-activated breast carcinoma cells (Kubo et al., 2004). GLI inhibitors have also been extensively developed for clinical trials (Bhateja et al., 2019; Riobo-Del Galdo et al., 2019).

### Fibroblast Growth Factor Signaling

The fibroblast growth factor (FGF) family is composed of at least 22 members and 4 FGF receptors (FGFRs) that are involved in various aspects of vertebrate development (Pownall and Isaacs, 2010). Upon ligand binding, FGFRs dimerize and become phosphorylated in the intracellular tyrosine kinase domains, which, in turn, leads to the activation of various downstream proteins; e.g., FGFR substrate 2 (FRS2), phospholipase Cγ (PLCγ), among others. FGF signals are typically transduced by the RAS/MAPK, PI3K/AKT, or PLCγ downstream cascades which regulate a myriad of cellular processes like cell growth. Given its wide-ranging effects, FGFR signaling is tightly regulated, exemplified by its negative regulation by MAPK phosphatase 3, Sprouty proteins, and similar expression to FGF (SeF) family members (Santolla and Maggiolini, 2020) (Figure 3B).

FGF10 and its main receptor, FGFR2B are involved in epithelial-mesenchymal interactions, as suggested by their complementary expression domains in the MRs. At E10.5, *Fgf10* is expressed in the ventral region of the thoracic somites (Mailleux et al., 2002; Veltmaat et al., 2006). From E11 to E12, *Fgfr2b* is expressed in the mammary epithelium (Spencer-Dene et al., 2001; Mailleux et al., 2002). Strikingly, *Fgf10* ^*−/−*^ embryos only form MR4 (Mailleux et al., 2002; Veltmaat et al., 2006). Even so, development of MR4 proceeds aberrantly as at E18.5, *Fgf10* ^*−/−*^ MR4 consists only of a sprout connected to the nipple, demonstrating the involvement of *Fgf10* in branching morphogenesis in addition to MR induction. The requirement of FGF signaling in branching morphogenesis is consistent with the finding that at E18.5, all 4 FGFRs (most prominently FGFR2 and FGFR3) are expressed in the MRs (Spike et al., 2012). Like *Fgf10* ^*−/−*^ embryos, *Fgfr2b* ^*−/−*^ embryos only induce MR4 (Veltmaat et al., 2006), albeit transiently as it regresses by E13 due to mammary epithelial apoptosis. Intriguingly, high levels of FGF10 are required for MR3 induction as *Fgf10* ^*mlcv/-*^ (*Fgf10* hypomorph) embryos lack MR3 (Veltmaat et al., 2006). This suggests MR-specific requirement for FGF signaling activation.

*Fgf7*, another ligand of *Fgfr2b*, is expressed at E12.5 in the mesenchyme, before the formation of the mammary mesenchyme. This suggests that FGF7 may act redundantly with FGF10 to activate FGFR2B for MR4 maintenance. By E15.5, *Fgf7* expression decreases but expands into the adjacent fat pad precursor (Mailleux et al., 2002). Other ligands of FGFR2B namely *Fgf1* and *Fgf3* are not expressed in the MRs.

FGF signaling misregulation is common across all breast cancer subtypes (Santolla and Maggiolini, 2020). FGFR1 amplification is the most frequent genomic alteration whereas FGFR2-4 amplification is relatively less common in breast cancer (Reis-Filho 2006). Interestingly, FGFR1 amplification may be breast cancer subtype-specific: in particular, the FGFR1 locus (8q12) is amplified in nearly 15% of hormone receptor-positive breast cancers but only in 5% of triple-negative breast cancers (TNBC). Aberrant activation of FGF/FGFR signaling caused by FGF overexpression or FGFR1 amplification and overexpression in ER^+^ breast cancer cells is associated with estrogen-independent cell proliferation, metastasis and reduced distant metastasis-free survival (McLeskey et al., 1998; Turner and Grose, 2010). These studies, and many others, strongly suggest the involvement of FGF signaling in malignant progression. As such, various therapeutic approaches have been developed to inhibit the pathway including small molecule inhibitors and monoclonal antibodies (mAbs) that block FGFRs or entrap FGFs (Santolla and Maggiolini, 2020).

### P190b RhoGTPase Activating Protein, Insulin Receptor Substrate, and Insulin-Like Growth Factor 1 Receptor Signaling

*P190b* is a member of the RhoGAP family which are negative regulators of RhoGTPase activity (Burbelo et al., 1995). At E12.5, *p190b* is expressed in the mammary epithelium while at E14.5 *p190b* expands its expression domain, but at a lower level, to the adjacent mesenchyme (Heckman et al., 2007). *P190b* ^*−/−*^ embryos develop hypoplastic MRs with disorganized mammary mesenchyme that lack AR expression (Heckman et al., 2007). Thus, *p190b* is required for MR growth, mammary mesenchyme specification, maturation and potentially sexual dimorphism. At E14.5, *p190b* ^*−/−*^ MRs show a decrease in the expression of adaptor proteins, insulin receptor substrate 1 (IRS1) and IRS2 and the insulin-like growth factor 1 receptor (IGF1R) signaling marker, phosphorylated Akt. IRS1 and IRS2 are expressed in the mammary epithelium and mesenchyme at E14.5*.* Similar to *p190b* ^*−/−*^ embryos, *Irs1* ^*−/−*^;*Irs2* ^*−/−*^ compound mutants develop hypoplastic MRs and lack mammary mesenchyme differentiation at E14.5 (Heckman et al., 2007). Finally, and consistent with the previous mutants, *Igf1r* ^*−/−*^ embryos develop hypoplastic MRs at E14.5. Taken together, P190B, IRS1, IRS2, and IGF1R form a signaling network that regulates various aspects of embryonic mammary development (Figure 3C).

Although RhoGTPases are commonly overexpressed and hyperactivated in breast cancers (Tang et al., 2008), paradoxically, *P190b* has been implicated as an oncogene in breast carcinogenesis. *P190b* haploinsufficiency inhibits MMTV-Neu tumor formation, progression, angiogenesis, and metastasis (Heckman-Stoddard et al., 2009). Consistently, specific overexpression of p190b in the mammary epithelium, also in MMTV-Neu mice, led to enhanced tumorigenesis and metastasis mediated by downstream Rac1-dependent reactive oxygen species (ROS) production (McHenry et al., 2010).

Despite their homology, IRS1 and IRS2, have distinct functions in regulating breast cancer progression (Gibson et al., 2007). Specifically, IRS2 is a positive regulator of metastasis (Nagle et al., 2004), whereas IRS1 is a suppressor of metastasis in the MMTV-PyMT mouse model (Ma et al., 2006). More recently, IRS1 has also been implicated in progesterone receptor (PR)-driven stemness and endocrine resistance in ER^+^ breast cancer (Dwyer et al., 2021). The IGF1R-IRS1/2 signaling axes may be important in breast cancers as at least 50% of breast tumors have activated IGF1R (Ekyalongo and Yee, 2017).

Several strategies have been developed to inhibit IGF1R signaling including the use of mAbs, small-molecule tyrosine kinase inhibitors of IGF1R and insulin receptor, and ligand neutralization. However, clinical trials show no appreciable benefit of these approaches thus far (Ekyalongo and Yee, 2017). A more promising approach uses the small-molecule tyrphostin, NT157 to target IRS; this method downregulates IRS protein expression and sensitizes ERα^+^ breast cancer cells to the mammalian target of rapamycin (mTOR) inhibitor, rapamycin. Moreover, NT157 inhibits the growth of tamoxifen-resistant ERα^+^ breast cancer cells (Yang et al., 2018).

### Parathyroid Hormone-Related Protein Signaling

Parathyroid hormone-related protein (PTHRP) derives its name from and shares structural and functional similarities with parathyroid hormone (PTH). PTHrP and PTH bind to and signal through the Type 1 PTH/PTHRP receptor (PTHR1) which is a G-protein coupled receptor. Activation of PTH1R causes an acute increase in intracellular signaling molecules including the two classical G protein signaling cascades initiated by adenylate cyclase (AC) and phospholipase C (PLC). This leads to a variety of responses including the transcription of target genes and the regulation of intracellular calcium levels (Hiremath and Wysolmerski, 2013) (Figure 3D).

The complementary expression pattern of *Pthrp* and *Pthr* in the embryonic mammary gland is indicative of their roles in mediating epithelial-mesenchymal interactions. *Pthrp* is expressed in the mammary epithelium from the placodal stage through birth, whereas *Pthr1* is expressed in the mammary and dermal mesenchyme (Wysolmerski et al., 1998). Disruption to either gene results in the induction of normal MRs, however nipple formation and subsequent development are impeded (Wysolmerski et al., 1998). *Pthrp* has non-cell autonomous roles as it is necessary for the differentiation and maturation of the mammary mesenchyme. In *Pthrp* ^*−/−*^ embryos, mesenchymal cells lack AR and tenascin C expression, although they continue to condense around the epithelial bud (Wysolmerski et al., 1998; Dunbar et al., 1999; Hiremath et al., 2012). The lack of AR expression causes the MRs of *Pthrp* ^*−/−*^ and *Pthr1* ^*−/−*^ male embryos to resist androgen-mediated destruction that is essential for sexual dimorphism (Dunbar et al., 1999). Conversely, overexpression of *Pthrp* under the control of the ectoderm- and MR-specific *Krt14* promoter results in aberrant mammary mesenchyme differentiation and supernumerary nipple formation in the ventral epidermis (Foley et al., 2001). Taken together, PTHRP-PTHR1 signaling is critical for epithelial-mesenchymal cross-talks, sexual dimorphism, mesenchymal maturation, and branching morphogenesis.

*Pthrp* upregulates bone morphogenetic protein receptor 1a (*Bmpr1a*) expression in the mammary mesenchyme to enable BMP4-mediated signaling. Ductal outgrowth regulated by PTHrP may be mediated in part by BMP4 as its supplementation can rescue ductal outgrowth defects in *Pthrp* ^*−/−*^ MRs. This points to critical roles of BMP signaling in the mesenchyme and in the development of the MRs after E12.5 (Hens et al., 2007) (see section on BMP signaling). The specification of the mammary mesenchyme may also implicate the homeobox transcription factor *Msx2* (Hens et al., 2007) (see also section on HOX transcription factors) and canonical WNT signaling in the mesenchyme, as its expression and activity, respectively, are dependent on *Pthrp* (Hiremath et al., 2012).

PTHRP and PTHR1 are often overexpressed in breast cancer and notably, PTHR1 is commonly overexpressed in breast cancer stroma (Henderson et al., 2006). Genome-wide association studies have implicated both the parathyroid hormone-like hormone (PTHLH) and the PTHR1 loci as breast cancer susceptibility genes (Garcia-Closas et al., 2013; Michailidou et al., 2013). In line with this, loss of *Pthrp* in the mammary epithelium of the MMTV-PyMT mice delays tumor initiation, progression, and reduces metastasis. Besides regulating genes involved in cell proliferation, angiogenesis, and apoptosis, PTHRP also affects the expression of adhesion factor CXCR4, which may be crucial for metastatic dissemination (Li et al., 2011).

PTHRP is important for metastatic colonisation in various distal sites, in particular the bone through the upregulation of osteoblastic receptor activator of NF-κB ligand (RANKL) which drives bone destruction, and downregulation of osteoprotegerin (OPG) expression (Guise et al., 2002). Consistent with this, a neutralising mAb against PTHRP diminished tumor growth and lytic bone lesions in MDA-MB-231 human breast cancer mice xenografts (Guise et al., 1996). PTHRP-specific neutralizing antibodies have also been shown to reduce lung metastases in MDA-MB-435 mice xenografts (Li et al., 2011), suggesting the broad utility of such antibodies and the feasibility of PTHRP as a target for metastasis.

### Bone Morphogenetic Protein Signaling

Bone morphogenetic proteins (BMPs) constitute a large family of secreted growth factors from the transforming growth factor beta (TGFβ) family of ligands that are involved in many aspects of development. The binding of BMPs to their cognate receptors results in the phosphorylation of the “Small”, receptor-regulated “Mothers Against Decapentaplegic” homolog (SMAD) family members, notably SMAD1, SMAD5, and SMAD 8. Phosphorylated SMAD 1/5/8 forms a complex with SMAD4 that translocates into the nucleus to regulate the expression of target genes (Chen et al., 2004; Wang et al., 2014) (Figure 3E).

*Bmp2* is expressed in the mammary epithelium at E13.5 (Phippard et al., 1996), although its function remains to be elucidated as *Bmp2* ^*−/−*^ mutants are not viable before the onset of mammogenesis. From E11.5 to E14.5 *Bmp4* is expressed predominantly in the mesenchyme and at lower levels in the mammary epithelium (Phippard et al., 1996; Hens et al., 2007). *Bmp4* is involved in various processes in the embryonic mammary gland including the positioning of the mammary line along the dorso-ventral axis, along with *Tbx3*, at E11.5 (Cho et al., 2006), epithelial-mesenchymal maturation and ductal outgrowth in conjunction with its receptor, *Bmpr1a* (Hens et al., 2007)*. Bmpr1a* expression in the mesenchyme is regulated by PTHRP (see section on PTHRP signaling).

Aberrant expression of BMPs and misregulation of BMP signaling has been associated with breast cancer; however, their roles and effects in tumorigenesis can be context-dependent and ligand-specific. Several BMPs, including BMP2, BMP6, BMP9, BMP10, BMP15, and GDF9a inhibit the proliferation of breast cancer cells, whereas BMP4 and BMP7 can either promote or inhibit proliferation in different contexts (Alarmo and Kallioniemi, 2010; Zabkiewicz et al., 2017).

Besides proliferation, BMPs can also regulate cancer cell stemness. Recombinant BMP2 induces EMT and stemness of breast cancer cells *via* the Rb and CD44 signaling pathways, which leads to metastasis (Huang et al., 2017). In contrast, BMP4 may act as an autocrine mediator to activate SMAD7 and block metastasis in animal models of breast cancer. Restored BMP4 expression or therapeutically administered BMP4 protein sensitizes cancer cells to anoikis, reduces the number of circulating tumor cells and the extent of metastasis, thereby resulting in increased survival (Eckhardt et al., 2020).

Various approaches have been developed to modulate BMP signaling, including the use of BMPR kinase inhibitors and other soluble decoy receptors which can prevent the interaction of BMPs in the extracellular space with membrane-embedded receptors. Downregulation of SMAD signaling *via* the silencing of the E3 ubiquitin ligase, Smurf1 also attenuates BMP signaling (Lowery and Rosen, 2018).

### Ectodysplasin Signaling

Ectodysplasin (*Eda*) is a member of the tumor necrosis factor (TNF) ligand superfamily, and functions with its receptor, *Edar*, to regulate the development of a variety of ectodermal appendages (Figure 3F). Upon EDA binding, EDAR recruits the adaptor protein EDARADD and *via* TRAF6 activates the IKK complex. This leads to the phosphorylation of I-κB, translocation of NF-κB into the nucleus and target gene transcription (Mikkola and Thesleff, 2003) (Figure 3F).

*Edar* is expressed in the mammary epithelium at E12 (Pispa et al., 2003) whereas *Eda* is expressed in the mesenchyme before and during branching morphogenesis from E15.5 to E17.5 (Voutilainen et al., 2012). Although *Eda* ^*−/−*^ (Tabby) mutants have normal numbers of mammary glands, the glands have smaller ductal trees with fewer branches (Mustonen et al., 2003). Conversely, overexpression of *Eda* using the *Krt14* promoter induces supernumerary MRs which develop into mature glands in the adult, between MR3 and MR4 (Mustonen et al., 2003; Mustonen et al., 2004). *Krt14-Eda* MRs display precocious branching morphogenesis and ectopically activate NF-κB. EDA signaling is likely mediated by NF-κB as inhibition of NF-κB, concurrent with *Eda* overexpression result in smaller ductal trees with fewer branches at E18 (Voutilainen et al., 2012). Important regulators of embryonic mammary gland development namely *PTHrP*, *Wnt10a*, *Wnt10b* and other genes such as the epidermal growth factor (EGF) family ligands, *Areg* and *Epgn*, have been identified as potential transcriptional targets of EDA/NF-κB signaling during ductal development, suggesting the integration of these pathways for MR development.

*Krt14-Eda* mice do not show palpable tumors, however, elevated EDAR signaling in *Edar* ^*Tg951*^ (*Edar* copy-number amplification) transgenic mice results in a high incidence of mammary tumors in breeding female mice. These tumors may bear important, clinical-relevant characteristics as they resemble *EDAR*-high human tumors which lack ER expression but have elevated β-catenin transcriptional activity and extensive squamous metaplasia (Williams et al., 2022).

### Neuregulin 3

The neuregulin (Nrg) family consists of four genes, *Nrg1*, *Nrg2*, *Nrg3*, and *Nrg4*, which is characterized by a conserved domain related to the EGF family of ligands. *Nrg3* is a ligand for the receptor tyrosine kinase erythroblastic leukaemia viral oncogene homolog 4 (ERBB4) that belongs to the ErbB receptor tyrosine kinase family (Zhang et al., 1997). Ligand binding causes the receptor to dimerize and activate intracellular tyrosine kinase domain, leading to the activation of downstream signaling cascades such as the PI3K/AKT and MAPK pathways to regulate various processes (Stern, 2000; Hayes and Gullick, 2008) (Figure 3G).

In embryonic mammary gland development, *Nrg3* is likely involved in early inductive events as it is expressed in the dermal mesenchyme adjacent to the presumptive mammary region and the presumptive mammary region itself at E11 (Howard et al., 2005). Subsequently, *Nrg3* is expressed in the mammary epithelium. Like *Nrg3*, *Erbb4* is expressed in the dermal mesenchyme underlying the presumptive MR3 and MR4 at E11.5. Subsequently, *Erbb4* is expressed in the mammary epithelium and ectoderm at E12.5 and E13 (Howard et al., 2005).

Scaramanga (*Nrg3* ^*ska*^, *Nrg3* hypomorph) embryos often fail to induce MR3 but induce supernumerary MRs adjacent to the site of MR4, suggesting the anatomical region-specific roles of *Nrg3* in mammogenesis. Application of recombinant NRG3 or *Nrg3* overexpression using the *Krt14* promoter induces MRs along the mammary line (Howard et al., 2005; Panchal et al., 2007). *Nrg3* ^*ska*^ MR3 display defects in mammary mesenchyme specification characterized by the downregulation of *Lef1*, *ER*, *AR*, and *Pth1r* expressions at E12.5 (Kogata et al., 2014).

In human breast cancer cell lines, NRG3 activates ectopically-expressed ERBB receptors (ERBB1-4). Whereas NRG3 is potentially overexpressed in breast cancer, paradoxically, recombinant NRG3 diminished the growth of human breast cancer cells *in vitro*. These results indicate potential dose-dependent effects of NRG3 (Hijazi et al., 1998). On the other hand, ERBB family receptor tyrosine kinases are commonly overexpressed in breast cancers, in particular, *ErbB2* or HER2/neu amplification constitute a major breast cancer subtype found in 15–30% of breast cancers while *ErbB4* overexpression is less common. Intriguingly, *ErbB4* has context-dependent tumor suppressive and oncogenic roles (Sundvall et al., 2008). Therapeutics targeting ERBB receptors in breast cancer include the humanized anti-ErbB2 antibody trastuzumab (Herceptin) and the tyrosine kinase inhibitor, lapatinib which are efficacious and widely used in the clinic.

### NOTCH Signaling

The NOTCH pathway mediates juxtacrine cellular signaling where transmembrane ligands on one cell activate transmembrane receptors on a juxtaposed cell (Hori et al., 2013; Siebel and Lendahl, 2017). Four receptors (NOTCH1–4) and five ligands—Delta-like ligand 1, 3, 4 (DLL1, 3, 4), Jagged 1 and 2 (JAG1, 2)—have been described in mammals (Hori et al., 2013) (Figure 3H).

NOTCH signaling is activated upon the binding a NOTCH ligand to its receptor, which triggers receptor cleavage by a member of the disintegrin and metalloprotease domain family (ADAM17 or ADAM10) and a presenilin-dependent γ-secretase complex. The cleaved intracellular domain of the NOTCH receptor (NICD) translocates into the nucleus where it forms a complex with the DNA-binding protein, CSL, and other transcriptional co-activators to drive Notch-target genes expression (Kopan and Ilagan, 2009; Hori et al., 2013).

Several *in vivo* lineage tracing studies demonstrate that at the population level, embryonic mammary gland cells are multipotent, bearing the capacity to give rise to basal and luminal cell lineages in the postnatal mammary gland (Van Keymeulen et al., 2011; Wuidart et al., 2016). However, NOTCH1 activation *via* the transgenic overexpression of NOTCH1 intracellular domain (N1ICD)—a ligand-independent active form of the NOTCH1 receptor—imposes a luminal ERα^−^ cell fate onto E13.5 cells (Lilja et al., 2018). This suggests that NOTCH signaling must be inactivated to maintain the multipotency of the embryonic mammary cells (see section on cellular lineages and stem cell potency).

NOTCH signaling is frequently deregulated in different breast cancer subtypes and is associated with the acquisition of therapeutic resistance (Lamy et al., 2017; Krishna et al., 2019; Nandi and Chakrabarti, 2020). Overexpression of the NOTCH1 intracellular domain with the MMTV promoter [MMTV-Notch1 (intra)] impairs mammary gland development and induces mammary tumors, suggesting the oncogenic role of NOTCH1 (Hu et al., 2006). Other studies also show NOTCH1 activation and its association with metastatic breast cancer cells (Mohammadi-Yeganeh et al., 2015). Interestingly, accumulating evidence points to the involvement of juxtacrine NOTCH signaling between tumor cells and cells that constitute the tumor microenvironment such as immune cells, cancer associated fibroblasts and endothelial cells to promote malignant progression (Meurette and Mehlen, 2018).

Gamma-secretase inhibitors (GSIs) are pan-NOTCH inhibitors that are the first and most extensively explored small molecules targeting the NOTCH pathway. GSIs reduce the levels of NICDs and several other substrate proteins, thereby inhibiting downstream signaling. Other promising approaches include anti-Notch mAbs which target the receptor-ligand binding domain or the negative regulatory region (NRR) of the NOTCH receptor, and, in turn, block intracellular NOTCH cleavage by γ-secretases and signal transduction (Lamy et al., 2017).

### Homeobox Transcription Factors

Homeotic (*Hox*) genes encode for the prototypic homeobox transcription factors, which are known to be master regulators of developmental programs (Carroll, 1995). The role of *Hox* genes in regional specification is reflected in their sequential, partially overlapping expression domains along the antero-posterior body axis. This is also reflected in the relative positions of the *Hox* genes on the chromosome (Morgan, 1997).

Several homeobox factors are involved in MR development: *Hoxc6* ^*−/−*^ mouse embryos form hypoplastic thoracic MRs at E12.5 but their inguinal MRs are unaffected (Garcia-Gasca and Spyropoulos, 2000). The position-specific phenotype is consistent with the expression of *Hoxc6* in the anterior somites underlying the thoracic MRs, and its absence in the posterior somites underlying inguinal MRs. *Hoxb9* and *Hoxd9* are expressed in the mammary mesenchyme at E12.5 (Chen and Capecchi, 1999), although their function, if any, in embryonic mammary gland development is unknown. Supernumerary mammary gland formation has been attributed to the ectopic expression of *Hox* genes (Schmidt, 1998). Taken together, *Hox* genes are important for MR-specific induction and growth.

The *Msx* genes belong to a small family of three homeobox-containing transcription factors related to the muscle segment homeobox gene, *msh*, in Drosophila (Davidson, 1995). *Msx1* and *Msx2* are expressed in the mammary epithelium at E13.5 (Phippard et al., 1996) whereas at E14.5, *Msx2* is expressed in the mesenchyme (Satokata et al., 2000). *Msx1* ^*−/−*^ MRs develop normally (Phippard et al., 1996) while in *Msx2* ^*−/−*^, MR4 development arrests at the sprout stage by E16.5. *Msx1* ^*−/−*^;*Msx2* ^*−/−*^ compound mutants form a hypoplastic, protruded MR4 coincident with defective mammary mesenchyme at E15.5. These aberrant phenotypes are linked to the loss of *Lef1* expression in the epithelium and mesenchyme (Satokata et al., 2000). Thus, *Msx1* and *Msx2* play non-redundant roles in MR development. Lastly, the paired-box homeobox gene 3 (*Pax3*) is expressed in the somites at E11.5 (Veltmaat et al., 2006). *Pax3* ^*ILZ/ILZ*^ (*Pax3* null) mouse embryos do not form hypaxial buds of the somites and show delayed induction of MR3; this finding highlights the role of the somites for MR3 induction (Veltmaat et al., 2006).

*Hox* transcription factors play multiple roles in breast cancer including cell cycle control, apoptosis, angiogenesis and cell-cell communication (Lewis, 2000; Briegel, 2006). HOXB7 has been reported as an oncogene associated with the upregulation of bFGF expression (Care et al., 1998) and EMT induction (Wu et al., 2006). Bisphenol-A, a known endocrine-disrupting compound that increases the risk of breast cancer, induces the expression of estrogen-regulated *Hoxc6* (Hussain et al., 2015) and *Hoxb9* (Deb et al., 2016). Comparatively, other *Hox* family members like HOXA9 may exhibit tumor suppressive roles by upregulating the expression of BRCA1 in breast cancer cells. Moreover, HOXA9 downregulation is associated with elevated tumor invasion, metastasis, and patient mortality (Gilbert et al., 2010). *Hox* genes may engage epigenetic regulators to regulate tumorigenesis and metastasis (Sun et al., 2013).

One potential strategy to inhibit HOX function is *via* the use of HXR9 peptides which prevents HOX binding to PBX, a transcription co-activator common to many HOX proteins. HXR9 causes apoptosis in multiple breast cancer cell lines (Morgan et al., 2012). Overcoming the functional redundancies among the different HOX family members is one of the main challenges for HOX-based therapeutic strategies (Feng et al., 2021).

### T-Box Transcription Factors

The T-box family (TBX) are transcriptional activators or repressors that are defined by a highly conserved T-box DNA binding domain (Wilson and Conlon, 2002). *Tbx2* is expressed in the mammary mesenchyme at E11.5 while *Tbx3* is expressed in the mammary epithelium between E11.5 and E12.5 (Chapman et al., 1996; Eblaghie et al., 2004). *Tbx3* expression is restricted to the dorsal domain by ventral *Bmp4* expression, which also determines the position of the mammary line (Cho et al., 2006). *Tbx3* ^*tm1Pa*/+^ (*Tbx3* heterozygous) mice form mammary placodes but maintenance of a subset of thoracic buds, nipple formation and ductal branching are impaired (Jerome-Majewska et al., 2005). Expectedly, *Tbx3* ^*tm1Pa*^/^*tm1Pa*^ (*Tbx3* null) embryos have a more severe phenotype, failing to induce most MRs altogether (Davenport et al., 2003). *Tbx2* ^*tm1Pa/+*^ (*Tbx2* heterozygous) embryos form MRs normally whereas *Tbx2* ^*tm1Pa/tm1Pa*^ (*Tbx2* null) embryos show inductive defects for MR2 and MR5.

Although induction is largely not affected in *Tbx2* ^*+/−*^ *;Tbx3* ^*+/−*^ compound mutants, some thoracic MRs regress by E18.5. For MRs that do progress, nipple formation and branching morphogenesis are frequently affected (Jerome-Majewska et al., 2005). In sum, *Tbx* genes play important roles in the induction, maintenance, nipple formation and branching morphogenesis of the MRs.

Both TBX2 and TBX3 are deregulated in breast cancers. Interestingly, TBX2 is found in a region of amplification on chromosome 17q23, which is common to about 20% of human breast cancers (Sinclair et al., 2003). TBX2 may be involved in malignant progression as its overexpression correlates with advanced tumor stages and with aggressive, hereditary BRCA1/2 breast cancers. Mechanistically, the deregulation of TBX2 or TBX3 may result in the bypass of P53-mediated senescence, growth arrest and apoptosis in breast cancers. TBX2 and TBX3 suppress *Cdnk2a/p19Arf* (*p14Arf* in human) transcription, which induces cell cycle arrest at the G1 and G2 phase by interfering with MDM2, a negative regulator of P53 (Briegel, 2006). The repression of *p14Arf* by TBX3 overexpression may be mediated by HDACs (Yarosh et al., 2008). Interestingly, estrogen signaling expands breast CSCs in MCF7 breast cancer cells through a paracrine FGF/FGFR/TBX3 signaling pathway, suggesting a role for *Tbx3* in promoting stemness (Fillmore et al., 2010). On the other hand, *Tbx2* has been shown to repress the expression of *p21WAF1/CIP1*, a P53 target necessary for P53-mediated growth arrest (Prince et al., 2004). TBX2 overexpression directly represses E-cadherin transcription and promotes EMT (Wang et al., 2012). Importantly, TBX2 and TBX3 may additionally play non-redundant roles in breast cancers. For example, TBX2, but not TBX3, is associated with increased metastatic potential of breast tumors through its regulation of adhesion molecules like cadherins and integrins (Rowley et al., 2004). Therapeutic strategies for TBX proteins, currently unavailable, could be directed towards their unique small repression domains (Chang et al., 2016).

### GATA3

GATA3 belongs to a family of zinc finger transcription factors that bind to a consensus DNA sequence (A/T)GATA (A/G) in gene promoter regions to directly activate or repress target gene expression (Du et al., 2015).

GATA3 is expressed in the mammary epithelium and ectoderm at E12.5 (Asselin-Labat et al., 2007). Conditional deletion of *Gata3* in these compartments under the *Krt14* promoter (*Krt14–Cre*;*Gata-3* ^*flox/flox*^) results in the lack of induction and hypoplasia of a variable subset of MRs, as assessed by *Lef1* expression at E11.75 (Asselin-Labat et al., 2007).

GATA3 is one of the most frequently mutated genes in breast cancer and has context-dependent tumor suppressive or oncogenic roles. GATA3 heterozygosity in MMTV-PyMT mice expands the CD14^+^ c-kit^−/lo^ and c-kit^+^ luminal progenitor cell population and promotes tumorigenesis; contrastingly, overexpression of GATA3 in the same mouse model promotes cellular differentiation, reduces angiogenesis and inhibits tumorigenesis (Asselin-Labat et al., 2011). In MCF7 human breast cancer cells, GATA3, through its transcription regulation of CCND1 and in association with PARP1 promotes cell proliferation and tumorigenesis by facilitating the G_1_ to S phase transition in the cell cycle. *In vivo* studies further show that GATA3 knockdown dramatically reduces tumor volume (Shan et al., 2014).

In the context of metastasis, GATA3 overexpression in LM2-4175 breast cancer cell line, an aggressive derivative of MDA-MB-231, inhibits cancer cell expansion in the lung. This is linked to GATA3-mediated downregulation of ID1/-3, KRTHB1, LY6E and RARRES3 as well as upregulation of genetic inhibitors of lung metastasis such as deleted in liver cancer 1 (DLC1) and progestagen-associated endometrial protein, PAEP (Dydensborg et al., 2009). GATA3 may also suppress metastasis *via* the reversal of EMT (Yan et al., 2010).

### P63

The *p63* gene, a homologue of the tumor suppressor *p53*, is highly expressed in the basal or progenitor layers of epithelial tissues. Very strikingly, *p63* ^*−/−*^ embryos fail to induce all MRs and all other ectodermal appendages (Mills et al., 1999; Yang et al., 1999).

P63 has multifaceted roles in breast cancer (Gatti et al., 2019). Notably, the overexpression of *H-Ras* ^*V12*^ or *PIK3CA* ^*H1047R*^ oncogenes in MCF10A and MCF12A normal breast cell lines downregulates the expression of *∆Np63*, a *p63* isoform, which leads to EMT, increased cell motility, and invasiveness. Importantly, silencing of *∆Np63* alone induces EMT and recapitulates the pro-migratory action of these oncogenes; highlighting *∆Np63* as a critical effector (Yoh et al., 2016). The invasive properties of *p63* may be mediated through its target gene, membrane-type 1 membrane-anchored matrix metalloproteinase (MT1-MMP), a protease that is upregulated in *p63*-high basal breast cancers (Lodillinsky et al., 2016).

*∆Np63* is also involved in controlling the self-renewal potential and expansion of mammary CSCs. Downregulation of *p63* in MMTV-ErbB2-derived mammospheres significantly limits its self-renewal capacity *in vitro* and delays tumor growth *in vivo*. At the molecular level, *∆Np63* enhances HH signaling by directing the expression of SHH, GLI2, and PTCH1 (Memmi et al., 2015).

### Hormone Signaling

The steroid hormones estrogen and progesterone are essential for the development and function of the breast, bone as well as the reproductive and cardiovascular systems. Classically, estrogen or progesterone binding to their cognate nuclear receptors leads to receptor dimerization, nuclear translocation and binding to DNA response elements to activate or inhibit target gene expression (Fuentes and Silveyra, 2019) (Figure 3I).

Hormone signaling is essential for sexual dimorphism, a process where MR development diverges between the two sexes in the embryonic mouse MRs at E14.5. In males, testosterone acts on the AR-expressing mesenchyme to constrict the mammary epithelium which halts further development (Durnberger and Kratochwil, 1980; Sakakura et al., 1982). When AR expression is absent or downregulated, MR development proceeds aberrantly (see sections on BMP, PTHRP, P190B RHOGAP, IRS, and IGF1R signaling).

Hormone receptor (ER or PR)-positive breast cancers constitute the major proportion of breast cancer subtypes. The transcriptional activity of ERα is regulated by its post-translational modifications and the action of nuclear receptor co-regulators, which may contribute to the development of breast cancer (Manavathi et al., 2013). The cross-talk of ER with other steroid receptors like PR affects tumor progression. Notably, progesterone may enhance the anti-proliferative effect of standard anti-estrogen therapy by influencing ER binding and its target gene transcription (Siersbaek et al., 2018).

ERα signaling has been implicated in metastasis. In ERα^+^ primary tumors, more than 80% of lymph node metastases, and 65–70% of overall distant metastases retain ERα expression. Moreover, ERα expression in tumors is also correlated with the development of bone and lung metastases (Saha Roy and Vadlamudi, 2012). Mechanistically, the bone tropism of metastatic breast cancer cells may be mediated by the interaction of ERα and the EMT transcription factor ZEB1, which have been shown to modulate ERα-mediated transcription induced by estrogen or cAMP signaling (Mohammadi Ghahhari et al., 2022). ERα knockdown in MCF7 breast cancer cells induces potent EMT and changes in the expression and activity of matrix macromolecules (Bouris et al., 2015). Functional cross-talks between estrogens and insulin/insulin-like growth factors (IIGFs)—by affecting the tumor microenvironment—may contribute to metastasis (Vella et al., 2020).

In the clinic, anti-estrogen therapy including selective ER modulators (SERMs), selective ER down-regulators (SERDs), and aromatase inhibitors (AIs) is the standard of care for patients with ERα^+^ breast cancers (Siersbaek et al., 2018).

Evidently, the preceding list of genes and signaling pathways have demonstrably clear, important roles in embryonic mammary gland development and often context-dependent roles in promoting breast cancer. GATA3 mutations are almost always associated with breast cancer compared to other cancers. Similarly, PTHRP signaling dysregulation is frequently implicated in breast cancer and bone metastasis. As a hormone-sensitive tissue, hormone signaling dysregulation has important implications for breast tissue. This, however does not imply that these genes and signaling pathways have exclusive roles or specific associations with breast cancer. Indeed, many are known to drive tumorigenesis in other tissues in different contexts. In the case of WNT signaling, loss-of-function mutations in APC were first implicated in the hereditary colon cancer syndrome, familial adenomatous polyposis whereas HH signaling dysregulation is predominantly known to drive basal cell carcinomas. These and examples of other cancers are listed in Table 1.

## Parallels of Properties and Molecular Signatures of Embryonic Cells in Breast Cancer

### Cellular Lineages and Stem Cell Potency

The cellular hierarchy and clonal dynamics of cells during pre- and postnatal mammary gland development have been greatly clarified by *in vivo* lineage tracing methodologies. Promoter specific-Cre models that activate the expression of a reporter gene encoding a fluorescent protein or *lacZ* enable the identification and facilitate the tracking of progenies from a defined parental cell (Kretzschmar and Watt, 2012).

By and large, *in vivo* lineage tracing studies with multiple promoter-Cre models suggest that during the initial stages of development, embryonic mammary cells are multipotent at the population level (Van Keymeulen et al., 2011; Rodilla et al., 2015; Trejo et al., 2017; Wuidart et al., 2018). Clonal analyses, where a small number of cells (<1%) is initially labelled, showed the progressive segregation of basal and luminal lineages and has revealed of unipotent luminal cells such as *Notch1* ^*pos*^ cells at E12.5 (Lilja et al., 2018). Other subsets of unipotent luminal cells include *Axin2*-expressing cells at E12.5 (Van Amerongen et al., 2012) and the zinc finger transcriptional repressor, *Blimp1*-expressing cells at E17.5 (Elias et al., 2017). During postnatal development, evidence for both unipotency and multipotency the mammary stem epithelia have been reported. While overwhelming evidence suggest the unipotency and lineage restriction of basal and luminal cells through *in vivo* lineage tracing (Van Keymeulen et al., 2011; de Visser et al., 2012; van Amerongen et al., 2012; Lafkas et al., 2013; Prater et al., 2014; Rodilla et al., 2015; Trejo et al., 2017; Lilja et al., 2018; Wuidart et al., 2018; Matsuo et al., 2022), rare bipotent basal cell clones expressing *Krt5*, *Krt14*, *Lgr5* (Rios et al., 2014), the WNT target, *Procr* (Wang et al., 2015) and the NOTCH ligand *Dll1*-expressing cells (Chakrabarti et al., 2018) have also been observed.

During postnatal development, it is postulated that luminal and basal cell interactions are crucial to maintain lineage fidelity. Specifically, cell-cell interactions are mediated by TNF which is secreted by luminal cells and restricts basal cell multipotency. Ablation of luminal cells in the adult mammary gland reactivates the multipotency of normally unipotent basal stem cells both *in vivo* and in organoid models. Bulk- and single-cell transcriptomic analyses reveal that after luminal cell ablation, basal cells activate a hybrid basal and luminal cell differentiation program that is reminiscent of the genetic program that regulates multipotency during embryonic development before giving rise to luminal cells. This multipotency program is mediated by the activation of NOTCH, WNT and EGFR and downregulation of the TNF signaling pathways. Therefore, NOTCH, WNT and EGFR pathway inactivation, or TNF pathway activation were able to inhibit regeneration-induced basal cell multipotency. This demonstrates that heterotypic communication between luminal and basal cells—tightly regulated by embryonic pathways such as NOTCH and WNT—is essential to maintain lineage fidelity and stem cell potency in mammary epithelial stem cells (Centonze et al., 2020). It is tempting to speculate that disruption of normal cell-cell interactions that lead to a reversion to a multipotent embryonic cell state may be an early event in tumorigenesis. Similar to lineage ablation, reprogramming of cell states by an oncogenic stimulus such as PIK3CA^H1047R^ results in the acquisition of multipotency of both basal and luminal cells in the postnatal mammary gland and the recapitulation of embryonic gene signatures (Wuidart et al., 2018). This, in turn, leads to the development of different breast cancer subtypes and the acquisition of tumor heterogeneity (Koren et al., 2015; Van Keymeulen et al., 2015).

### Embryonic Molecular Signatures in EMT, Stem Cells and Breast Cancer Subtypes

Expression profiles analyses of various human tumor types have revealed the enrichment patterns of gene sets associated with embryonic stem cell identity. In breast cancers, this embryonic stem cell-like signature is often associated with high-grade, basal subtype ER^−^ tumors, with poor clinical prognosis (Ben-Porath et al., 2008).

Several recent studies have harnessed the advancements of multi-omics technologies to investigate the presence of embryonic signatures in breast tumor models. Bulk transcriptomic analyses show that subsets of embryonic mammary epithelial signature at E12.5 are activated in mouse *Brca1* ^*−/−*^;*p53* ^*+/-*^ tumors and malignant human basal-like breast cancers. The signature is composed of genes that encode predominantly transcriptional regulators, notably *Hox* genes, cell cycle, and actin cytoskeleton components. There is also evidence that the embryonic signatures that are reactivated in cancers are subtype-specific. Embryonic gene subsets that include regulators of neuronal differentiation, transcription, and cell biosynthesis were enriched in non-basal-like tumor subtypes and repressed in basal-like tumors. Moreover, several embryonic genes showed significant upregulation in hormone receptor negative, and/or grade 3 breast cancers. Notably, the transcription factor, SOX11, a progenitor cell and lineage regulator of non-mammary cell types, is found to be highly expressed in some *Brca1* ^*−/−*^ mammary tumors. Cancer cells may also activate latent embryonic mesenchymal signatures to undergo EMT. A list of 25 genes—ATL3, B3GNT5, BCL11A, CDCA2, CHST2, CORO1C, DNM1L, DNMT3A, EPHA4, GPC2, HDGF, IGF2BP3, JMJD4, KIF20A, PROX1, PTDSS1, RPS6KA3, SLC16A13, SOX11, TCF7L2, TMEM38A, TMOD1, TRIB2, TTC9C, and UCHL1—were found in the 37-gene tumor-associated embryonic epithelial signature. This gene set could be further evaluated for their roles as putative regulators of EMT in breast cancers and potentially serve as new targets for therapeutic intervention in the future (Zvelebil et al., 2013).

There is evidence for significant molecular similarity of stem-like subpopulations of mammary cells which are enriched at E18.5 (Spike et al., 2012; Makarem et al., 2013; Trejo et al., 2017), to breast cancers. Specifically, the fetal mammary stem cell (fMaSC) signature was enriched among aggressive basal-like and Her2^+^ tumors. The co-expression of myoepithelial and luminal keratins as well as vimentin, which is characteristic of the fMaSC-like state, suggests that the reversion of cancer cells to an embryonic-like state resembling the fMaSC and/or fetal stroma (fSTR) compartments could be driven by partial EMT (Spike et al., 2012). On the other hand, a stem-like, quiescent, hormone-sensitive subpopulation of mammary gland cells which originate in the MRs, *Lgr5* ^*+*^ *Tspan8* ^*hi*^, has been shown to bear molecular features of claudin-low breast tumors (Fu et al., 2017).

In a follow-up to the Spike (2012) study using single-cell transcriptomic analyses, Her2^+^ tumors and basal-like tumors - but not the equally proliferative luminal B tumors—were found to frequently show enrichment of fMaSC-like metabolic profiles including glycolysis, the Krebs cycle and fatty acid oxidation. (Giraddi et al., 2018). This finding suggests that the re-emergence of embryonic metabolic programs could be less associated with the enhancement of tumor cell proliferation. Rather, other processes associated with tumorigenesis such as cell state changes, cellular plasticity, and lineage specification, could be directed by such embryonic programs (Ben-Porath et al., 2008).

In silico analyses comparing bulk TCGA tumor gene expression data to the Giraddi mouse developmental trajectory show that normal, luminal A, and luminal B tumors map most closely to mature adult cells whereas Her2^+^ tumors map to slightly more immature cells. Basal tumors, which harbour great molecular heterogeneity, spanned the pseudotime encompassing both embryonic and adult cells along the basal trajectory (Thong et al., 2020). In the same study, gene expression analyses revealed that normal human mammary (NM) cells aligned to adult mouse cells whereas NM cells, which are conditionally-reprogramed (CR) *in vitro* to promote stem-like features, aligned more closely with mouse embryonic cells across the pseudotime trajectory. In contrast to NM cells, CR cells also develop hybrid cell states, characterized by the co-expression of basal and luminal lineage markers or epithelial and mesenchymal markers that are associated with aggressive cancers. Taken together, these results suggests that the acquisition of embryonic programs converts cells into a stem-like state, presenting with characteristics typical of a more developmentally immature phenotype.

There is also evidence that metastatic cells may leverage on embryonic or more generic pluripotency programs to facilitate malignant progression. Single-cell analyses show that low-burden metastatic cells harbouring basal- or stem-like characteristics may exploit embryonic programs for self-renewal and maintenance by the upregulation of pluripotency genes POU5F1 (also known as OCT4) and SOX2 as well as classical EMT markers (Lawson et al., 2015).

## Embryonic Molecular Signatures and Their Clinical Relevance for Breast Cancer

**FIGURE 4:**
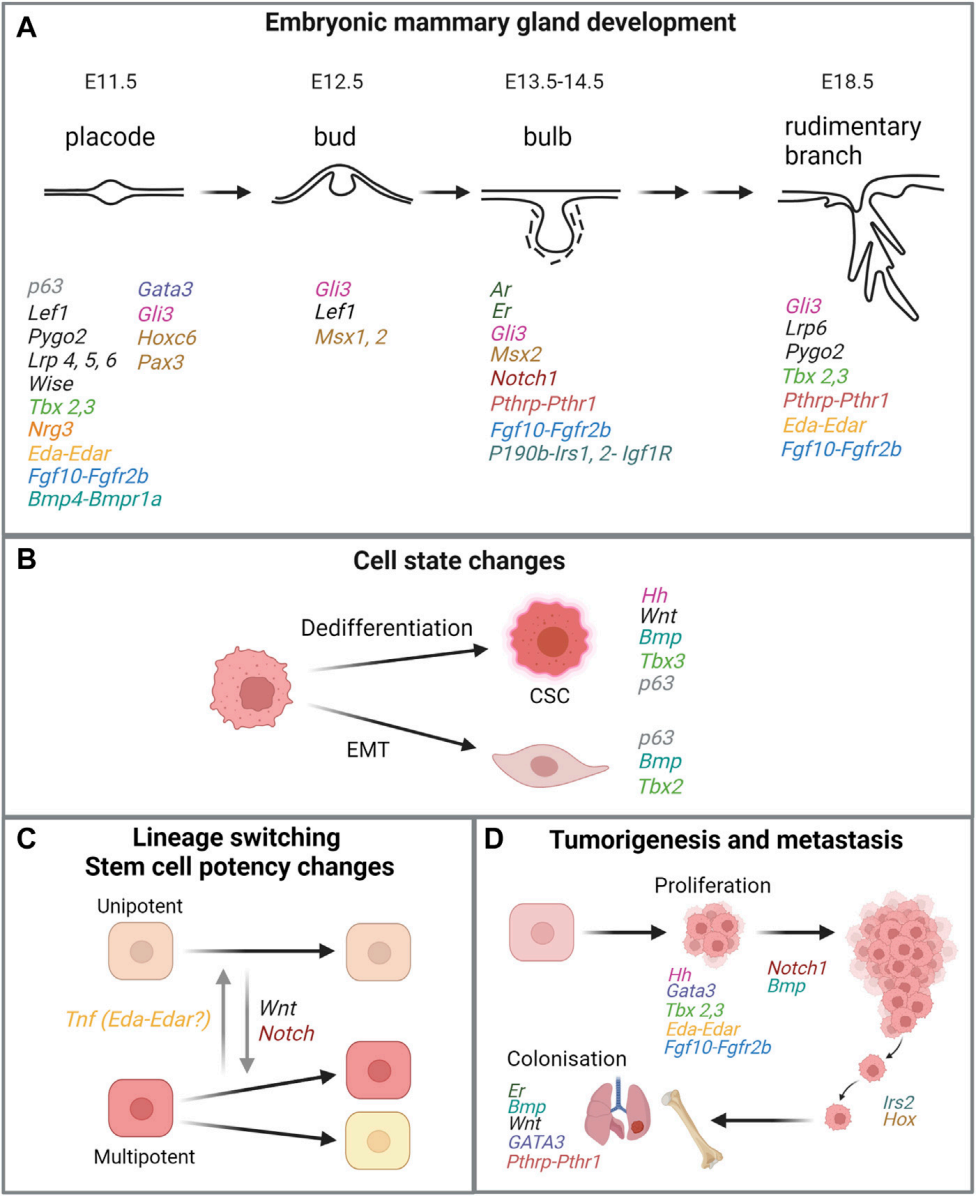
Similarities of genes or signaling pathways critical in embryonic mammary gland development and processes in breast cancer. **(A)** Key stages in embryonic mammary gland development and genes and their associated pathways involved in the respective stages. For MR-specific developmental processes that are regulated by relevant genes and signaling pathways, please refer to main text. **(B,C,D)** Processes that are linked to tumorigenesis and metastasis and mediated by the same genes or signaling pathways important for embryonic mammary gland development. Genes in the same signaling pathway are coded in the same colour. Figure created with BioRender.

Breast cancer and metastasis remain challenging problems globally. The striking similarities between embryonic progenitor cells to breast cancers and metastases could pave the way to pinpoint novel prognostic markers and targets for therapeutic intervention (Figure 4). It has been proposed that critical signaling pathways that promote pathogenesis may be masked by the over-representation of proliferation-, ER-, and Her2-related signaling signatures in many existing prognostic signatures. Therefore, these minor but critical signaling pathways may be uncovered by studying and distilling normal developmental paradigms such as the fMaSC and fSTR states. Such approaches could potentially deconvolute the complexities of tumor heterogeneity by narrowing the focus on subpopulations of cells that exhibit embryonic signatures and identifying targets specific for these populations.

There are some indications that show embryonic signatures may have clinically-relevant prognostic value. Only a very low percentage of patients who are treated with neoadjuvant chemotherapy will progress to pathological Complete Response (pCR). For this reason, novel approaches that can estimate the probability of pCR are highly desired. The predictive value of normal cellular expression features for pCR was evaluated using univariate and multivariate logistic regression analyses. Indeed, human luminal progenitors (LumProg) and mouse fMaSC expression features were identified as predictive of neoadjuvant chemotherapy efficacy across all breast cancer patients. These signatures were highly expressed in basal-like tumors, consistent with the clinical observation that basal-like tumors have better neoadjuvant chemotherapy response rates. On the other hand, benign luminal A and B tumors which are typically more resistant to neoadjuvant chemotherapy exhibit high expression of another MaSC signature subset (fMaSC-MmEnriched-feature2). Importantly, the prognostic value of these normal and embryonic signatures remained significant even after accounting for tumor intrinsic subtype, proliferative status, and other clinical parameters; in other words, normal cell signatures add information and prognostic value that are distinct from clinical features (Pfefferle et al., 2015). Along a similar vein, a prognostic gene expression signature derived from the E6.5 mouse which is representative of extensive cellular plasticity was shown to predict metastatic competence in human breast tumor cells (Soundararajan et al., 2015).

## Conclusion and Perspectives

The misregulation of genes and signaling that are important in regulating normal embryonic mammary gland development frequently occur in pathological conditions such as breast cancer (Howard and Ashworth, 2006). Thus, the identification and study of the mechanisms mediated by genes and signaling pathways during early development may give rise to deeper understanding of disease mechanisms. Notably, embryonic molecular signatures may complement conventional clinical parameters for the stratification of patients and to offer accurate predictions of outcomes among breast cancer patients.

To date, practical considerations and sample limitations have resulted in pooled mammary gland molecular profiling. Considering the unique genetic and developmental history of the mammary glands, such data pooling may have implications in the success of cancer research. Thus, it may be prudent to make MR-specific comparisons and analyses in future research.

Likewise, it will be of interest to further characterize and locate cells harbouring different cell states such as the hybrid state, reminiscent of embryonic cells, in the adult mammary gland and tumors using techniques such as spatial transcriptomics. With recent technical advancements in a suite of multi-omics methods, the identification of the metabolomic and epigenomic states of the embryonic mammary glands—and the parallels to breast cancers - will be exciting themes of research that will illuminate more insights into mammary gland development and cancer pathogenesis.

## Glossary

Mammary rudiment (MR): The mammary gland during embryogenesis.
Placode: Mammary rudiment at around E11.5 characterized by a multi-layered, disk-shaped morphology.
Genetically engineered mouse model (GEMM): Mouse (Mus musculus) with its genome modified using genetic engineering techniques. Genetic modifications may include gene loss-of-function (knockout, hypomorph) or gain-of-function (overexpression).
Sexual dimorphism: Developmental process in the E14.5 mouse where development diverges in male and female.
Epithelial-to-mesenchymal transition (EMT): A process that enables cancer cells to suppress their epithelial features and take on mesenchymal ones. As a result, cells detach from their primary site and migrate to distal sites.
Cancer stem cell (CSC): Subpopulation of cancer cells with tumor-generating and multi-lineage differentiation potential. These cells are commonly associated with chemoresistance.
MMTV-PyMT (mouse mammary tumor virus-Polyoma virus middle T antigen): Mouse mammary gland carcinoma model, which is characterized by rapid development of multifocal tumors and extensive lung metastasis.
Lineage tracing: A method to study the progenies of an initial population of cells that are marked genetically by a promoter-specific-Cre construct in combination with a reporter gene such as GFP.
